# Bioluminescence-Driven Optimization of Geminivirus-Based Vectors as Tools for Plant Biotechnology

**DOI:** 10.1021/acssynbio.5c00164

**Published:** 2025-08-04

**Authors:** Elena Garcia-Perez, Victor Vazquez-Vilriales, Marta Vazquez-Vilar, Araceli G. Castillo, Karen S. Sarkisyan, Rosa Lozano-Duran, Eduardo R. Bejarano, Diego Orzaez

**Affiliations:** † Instituto de Biología Molecular y Celular de Plantas, CSIC-UPV, Valencia 46022, Spain; ‡ Instituto de Hortofruticultura Subtropical y Mediterranea ’La Mayora’, Universidad de Málaga−Consejo Superior de Investigaciones Científicas (IHSM-UMA-CSIC), Universidad de Málaga, Málaga 29010, Spain; § Institute of Clinical Sciences, Faculty of Medicine and Imperial College Centre for Synthetic Biology, Imperial College London, London SW7 2AZ, U.K.; ∥ Centre for Plant Molecular Biology (ZMBP), Eberhard Karls University, Tübingen 72076, Germany

**Keywords:** plant synthetic biology, Nicotiana benthamiana, geminivirus, BeYDV, TYLCV, BCTV, bioluminescence

## Abstract

Viral replicons are valuable tools in plant biotechnology, widely utilized to increase recombinant protein production. Their ability to amplify gene dosage in a trigger-dependent manner also opens doors to regulatory applications. This work focuses on optimizing geminivirus-based vectors for Synthetic Biology applications in plants, using autobioluminescence as a sensitive, real-time reporter to characterize gene expression. Specifically, geminivirus-based synthetic replicons derived from bean yellow dwarf virus (BeYDV), tomato yellow leaf curl virus (TYLCV), and beet curly top virus (BCTV) were engineered and assessed for basal expression, inducibility, and recombinant protein coexpression potential. Our study provided insights into the strengths and limitations of each geminiviral replicon. BeYDV replicon displayed a robust activation profile suitable for complex tasks such as multigene expression, while TYLCV showed high expression levels despite moderate basal leakage. In contrast, BCTV demonstrated less favorable control and expression levels. Through a bioluminescence-based screening, the TYLCV system was further optimized to improve regulatory precision. These findings highlight the versatility of geminivirus replicons, paving the way for future engineering of synthetic gene circuits in plants.

## Graphical abstract

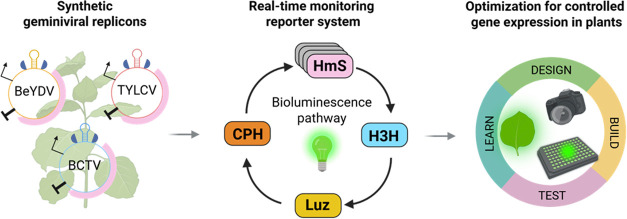

## Introduction

Virus-derived replicons have emerged as powerful tools in plant biotechnology due to their unique ability to hijack the cellular machinery to multiply designated pieces of genetic information.^1^ Among the different viral replicons employed in plant biotechnology, those derived from geminiviruses have gained particular attention due to their rapid and high-yield replication and wide flexibility for cargo insertion.^2^ The genetic amplification provided by geminiviruses replicons can serve different cellular bioengineering purposes, such as improving yields of biomanufactured products and increasing template concentration for gene editing.

Geminiviruses, members of the *Geminiviridae* family, are plant-infecting viruses with small (normally ∼3.0 kb) circular single-stranded DNA (ssDNA) genomes. These viruses infect a wide range of plants and are notorious for causing significant agricultural damage worldwide.^3,4^ Particularly, the three most prevalent and well-characterized geminivirus genera are Mastrevirus, Curtovirus, and Begomovirus. Geminiviruses multiply by repurposing the DNA replication machinery from the host cell to perform rolling-circle replication of the viral genome.^5^ In mastreviruses, this synthesis begins in a region known as the Short Intergenic Region (SIR), while in Begomovirus and Curtovirus, it starts near the Intergenic Region (IR), which is referred to as the Long Intergenic Region (LIR) in mastreviruses. The dsDNA intermediate then undergoes replication through rolling-circle replication (RCR) and recombination-dependent replication (RDR) assisted by DNA polymerase δ. The RCR is initiated by the virus-encoded Replication-associated protein (Rep). Rep binds to iteron sequences, which are specific cis-acting motifs located within the IR/LIR. Additionally, the IR/LIR harbors a GC-rich repeat inverted sequence forming a stem-loop structure that contains the highly conserved nonanucleotide 5′-TAATATTAC-3′. This nonanucleotide serves as the cleavage site for Rep, whose action provides a 3′–OH group in the dsDNA genome that can be extended by polymerase δ.^5^ The variability of iteron sequences between different geminivirus species plays a crucial role in determining virus-specific replication, while the Rep protein contains an iteron-related domain (IRD) that also confers specificity through DNA binding.^6,7^

Geminivirus-based synthetic replicons rely on two separate genetic elements, namely the Rep protein, and the replicative vector, where the gene of interest is flanked by intergenic regions.^8^ Mechanistically, the Rep protein leads to the circularization and rolling-circle replication of the vector by interacting with the flanking IR regions.^9^ Usually, both the Rep and the replicative vector into the plant cell are delivered into the plant cells transiently using *Agrobacterium*-mediated transient transformation.^10^ The bean yellow dwarf virus (BeYDV) from the genus Mastrevirus^11^ has served as the basis for most synthetic replicons developed to date using the agroinfiltration route. This approach has successfully produced large quantities of proteins of interest like vaccine candidates, antibodies, virus-like particles, and enzymes that mediate the heterologous enhanced expression of metabolites like triterpenoids. Moreover, the BeYDV replicative system has also been used for enhancing genome editing efficiency by the replication of the nuclease-edited DNA enzyme and the repair template in tobacco,^12^ potato,^13^ and tomato.^14^ The potential of BeYDV for enhancing protein expression is evident by the fact that very recently this system has been used in very divergent species like lettuce for the expression of virus-like particles and antibodies,^15^ pepper for the expression of capsanthin^16^ and microalgae to produce SARS-CoV-2 receptor antigens and basic fibroblast growth factor.^17^ Other geminiviruses used as the basis for the development of synthetic replicons include the beet curly top virus (BCTV), a Curtovirus with an exceptionally broad host range for replication.^18,19^ BCTV-derived replicative vectors have been used for expressing vaccine candidates in ,^20^ for plastid genome engineering in tobacco^21^ and for enhancing genome editing in by replication of CRISPR/Cas12a components.^22^ Also, the geminivirus wheat dwarf virus (WDV) has been used to create expression vectors that boost genome engineering in wheat^23^ and rice.^24^ Likewise, a sweet potato leaf curl virus (SPLCV) synthetic replicon was developed for genome editing in .^25^ Very recently, the geminiviral-based vector repertoire was expanded to include novel vectors based on tomato yellow leaf curl virus (TYLCV), honeysuckle yellow vein virus (HYVV), and mild curly top virus (BMCTV) for the expression of turbo green fluorescence protein (tGFP) in .^26^

Despite recent advances, the potential of geminiviral-based systems as tools for plant biotechnology remains largely unexploited. One of the most promising future directions is the development of genome-integrated inducible replicons. The rationale behind integrated replicons is that they remain silent until a user-defined signal activates replication via Rep expression. In that way, integrated replicons would keep all the advantages of the above-described *Agro*-delivered synthetic replicons, while circumventing the need for cumbersome transient expression and maintaining a high biosafety profile. Ideally, the availability of a battery of integration-competent geminiviral replicons derived from different phylogenetic origins would open the way not only to extend replicon technology to more crop species but also to combine two or more DNA amplification systems within the same plant. However, to date, maintaining effective control of replicon activation during the silent state remains a main challenge for the development of new integrated systems. Wild-type viral sequences are rich in cryptic regulatory sequences^27^ that could lead to a spurious activation of any derived synthetic replicon whose presence can only be determined experimentally.

Several geminiviral-based systems have been stably integrated into the plant genome,^7,28,29^ however to our knowledge only two are engineered to activate in response to chemical inducers: the INPACT system based on tomato yellow dwarf virus (TYDV),^30^ and the CuBe system previously developed in our laboratory and based on BeYDV.^31^ Both the INPACT and the CuBe systems enhance regulation by deconstructing the transcriptional unit of the gene of interest inserted in the geminiviral vector, preventing basal expression. In these systems, the promoter is only positioned upstream of the coding sequence once circularization occurs, a process mediated by the Rep protein. In the INPACT system, induction is triggered by ethanol via an ethanol-responsive promoter^32^ controlling Rep expression, while in the CuBe system, copper sulfate^33^ serves as the inducer for the same regulatory mechanism.

To broaden the range of geminiviral vectors for plant biotechnology uses, it is crucial to advance in viral deconstruction and to speed up the design-build-test-learn cycle,^34^ focusing on reducing basal expression. Viral deconstruction involves the definition of functional modules that can be easily exchanged and combined for biotechnological applications. Adaptation to modular cloning strategies, such as those developed by Dusel et al.^35^ for BeYDV and Neubauer et al.^36^ for BCTV, have provided more flexibility in assembling new geminiviral vectors. The adoption of a modular configuration facilitates not only synonymous exchange of DNA parts, but also novel rearrangement of those parts, enabling the exploration of optimized configurations that can be eventually adapted to a wider range of geminiviruses. Furthermore, achieving optimal configurations demands highly sensitive reporter systems capable of detecting minimal ‘leaky’ expression. Traditionally, geminiviral systems have relied on GFP or GUS reporters for characterization. Recently, a fungal bioluminescence pathway (FBP), composed of four genes, has emerged as a powerful alternative plant reporter system^37,38^ (Figure 1A). This pathway, which confers self-sustained light emission and therefore does not require the exogenous application of expensive luciferin substrate, is a cyclic metabolic process using caffeic acid, a common plant metabolite, as the starting substrate. The initial reaction, converting caffeic acid to hispidin, is catalyzed by a large (∼5.1 kilobases) polyketide synthase (HispS). HispS requires a posttranslational modification to function, typically achieved by coexpressing the phosphopantetheinyl-transferase like null pigmentation mutant A1 (npgA1) gene from in the plant.^37,38^ HispS has been identified as the transcriptional limiting step of the pathway.^39^ As an alternative to HispS, smaller hispidin synthases of plant origin that do not depend on post-translational modifications (HmS, ASCL, PKS2) can substitute for HispS, resulting in a plant-fungal Hybrid Bioluminescence Pathway (HBP).^40^ Benefiting from endogenous caffeoyl-CoA ligase activity present in plants, these smaller enzymes tap into the available pool of CoA-esters of hydroxycinnamic acids (Figure 1A). The FBP offers key advantages over traditional reporters like GFP, as it does not require external illumination, specific filter sets, or specialized imaging equipment and allows for imaging with standard consumer-grade cameras. This makes the FBP particularly suitable for noninvasive, real-time monitoring of gene expression in plant tissues. Recently, Calvache et al.^41^ developed a dual reporter system combining the luminescence of FBP with enhanced GFP (eGFP) fluorescence to correct for variations in transformation efficiency in transient expression in . This dual-output strategy ensures that autobioluminescence measurements are quantitatively reliable and not confounded by differences in transformation efficiency, thereby enhancing the robustness and reproducibility of transient expression assays.

**Figure 1:**
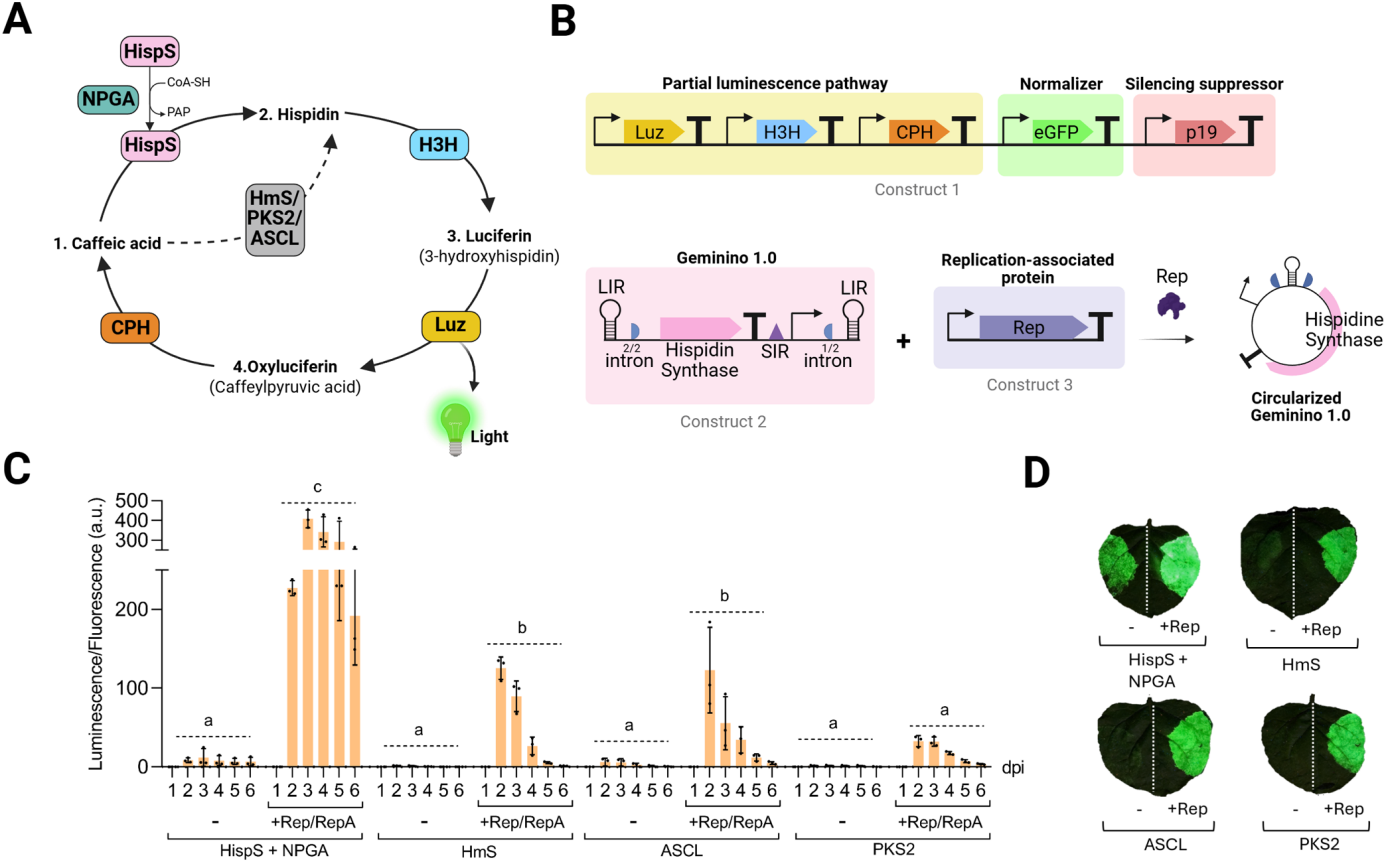
Evaluation of bioluminescence in leaves expressing different hispidin synthases through a BeYDV-based system. (A) Schematic representation of the bioluminescence pathway, illustrating the metabolic conversion of caffeic acid to hispidin and subsequent reactions leading to light emission. The pathway involves several enzymes, including the tested hispidin synthases (HispS, HmS, ASCL, and PKS2). (B) Schematic representation of the genetic constructs used in the study. Construct 1 contains constitutively expressed p35S-driven genes of the luminescence pathway (Luz, H3H, CPH), along with enhanced GFP (eGFP) and the p19 silencing suppressor. Construct 2 includes the geminiviral vector carrying different hispidin synthases (HispS/HmS/ASCL/PKS2). Construct 3 is a constitutive pNOS-driven polycistronic expression cassette for BeYDV Replication-associated proteins (Rep/RepA). These constructs were coinfiltrated into leaves to evaluate luminescence with and without Rep/RepA. In the HispS treatment, a 4th construct carrying p35S:NPGA was coexpressed. LIRs are the long intergenic regions (LIRs) from BeYDV, SIR is the short intergenic region, half circles are the initial (1/2 intron) and the final (2/2 intron) segments of a unique intron, T is the transcriptional terminator, and the arrow indicates the transcriptional promoter. (C) Luminescence normalized by fluorescence (luminescence/fluorescence arbitrary units) measured over six (1–6) days postinfiltration (dpi) for the different hispidin synthases (HispS + NPGA, HmS, ASCL, and PKS2) cloned in a BeYDV-based vector (Geminino 1.0) and coexpressed with (+) or without (−) Rep/RepA alongside the remaining genes of the bioluminescence pathway (H3H, Luz, CPH), the normalizer eGFP, and the silencing suppressor p19. Each bar represents the mean of three independent leaf discs, each collected from a different leaf of the same plant (*n* = 3). Error bars represent standard deviation. Statistical analysis was performed using one-way ANOVA with Tukey’s multiple comparisons test (*P*-value ≤ 0.05), comparing the area under the curve (AUC) of each time-course. Bars sharing the same letter are not significantly different. (D) Qualitative imaging of luminescence in whole agroinfiltrated leaves at 2 dpi for the same treatments described in (C). The figure includes images from Biorender (biorender.com).

In this work, we have employed a deconstruction approach, previously described for developing a modular BeYDV-based vector, to create new modular replicons based on TYLCV and BCTV. To facilitate this process and the subsequent optimization of new replicons, we have incorporated a new reporter system based on the HBP, showing the versatility of the new reporter to speed up replicon engineering in plants. As a result of several optimization steps, we obtained an improved version of a TYLCV-derived replicon showing a low basal expression and a highly enhanced dynamic range. By leveraging these viral systems, we aim to better regulate geminiviral vector replication to engineer gene circuits based on integrated replicons, and to understand the orthogonality of different geminiviral replicons when used in combination.

## Results

### Application of Bioluminescence Pathway as a Sensitive Qualitative and Quantitative Reporter for the Characterization of Geminiviral-Based Tools

To evaluate the potential of the autoluminescence pathway (Figure 1A) as a sensitive reporter system for characterizing geminiviral vectors, we cloned the hispidin synthase gene into the Geminino 1.0 vector based on BeYDV, as previously described^42^ (Figure 1B). The arrangement of the gene of interest in Geminino 1.0 was designed to harbor the 35S CaMV terminator and the 35S CaMV promoter downstream of the coding sequence (CDS) in a linear configuration. The gene of interest is flanked by the Long Intergenic Region (LIR) of BeYDV. These LIRs are recognized and nicked by the Replication-associated protein (Rep), which leads to DNA circularization. In this circular configuration, transcription can also proceed since the promoter relocates upstream of the CDS. Moreover, in this configuration, a split intron was engineered such that its first half lies upstream of the 3′ LIR and its second half downstream of the 5′ LIR in the linear prereplicon. Upon circularization, the LIR becomes flanked by the complete intron, which is then recognized and excised by the splicing machinery. This strategy ensures the removal of the LIR from the mature transcript, avoiding undesired secondary structures near the 5′UTR that could hinder translation initiation triggered from the circularized replicon. We tested different hispidin synthases—HispS, HmS, PKS2, and ASCL as candidates to incorporate into our reporter system. For luminescence assays, we used three T-DNA constructs: (i) geminiviral vectors containing HispS/HmS/PKS2/ASCL, (ii) constitutive 35S CaMV-driven genes for the rest of the pathway (Luz, H3H, CPH), eGFP normalizer, and p19 silencing suppressor, and (iii) constitutive pNOS-driven bicistronic BeYDV Replication-associated proteins (Rep/RepA) gene (Figure 1B). In the HispS treatment, a fourth construct carrying p35S:NPGA was coexpressed. These constructs were transiently coexpressed in leaves through agroinfiltration. Luminescence was monitored daily from one to 6 days postinfiltration (dpi) to analyze the effect of combining Geminino 1.0 with Rep/RepA. The normalized luminescence, expressed as luminescence/fluorescence ratios, showed that three of the tested hispidin synthases significantly increased luminescence in the presence of Rep/RepA, indicating the correlation between geminiviral replication and luminescence emission (Figure 1C). The combination of the fungal HispS and NPGA produced the highest luminescence, peaking on day 3. The plant-based synthases, HmS and ASCL, showed moderate increases, with maximum luminescence at day 2, while PKS2 had lower activity. When whole agroinfiltrated leaves were imaged at 2 days postinfiltration (dpi) (Figure 1D), HispS produced the highest luminescence, but it also exhibited strong baseline activity without Rep. Other hispidin synthases showed minimal luminescence in the absence of Rep, with HmS displaying more stable expression compared to ASCL, with less variation between leaves and greater consistency from day 2 to day 3. Steered by the simplicity of the system, we selected HmS as the preferred reporter gene for subsequent analysis of geminivirus-driven transcriptional activity: HmS is more compact than HispS, provides sufficient dynamic range, does not depend on NPGA, and probably, as a consequence, it reaches maximum expression levels at a shorter time point.

### Adaptation of TYLCV and BCTV Replicative Elements for Rep-Regulated Recombinant Protein Expression

To expand the toolbox of geminivirus-based replicative systems, we evaluated the potential of TYLCV and BCTV-based vectors as Rep-dependent systems for recombinant protein expression. Following a modular approach parallel to that used for BeYDV (Figure 1B), we adapted the replication machinery from TYLCV and BCTV genomes (Figure 2A) by using their intergenic regions (IRs) (Figure 2B) to develop their respective Geminino 1.0 type of vectors, along with their Replication-associated proteins (Rep). To test the functionality of these new modular systems, HmS was cloned into the Geminino 1.0 vectors, with luminescence used as a reporter. Constructs were transiently expressed in via agroinfiltration, coinfiltrated with or without the respective Rep for each geminivirus, as shown in Figure 1B. Normalized luminescence levels were measured over time to evaluate the performance of each system. As shown in Figure 2C, coinfiltration with Rep significantly increased luminescence in all three systems, with the TYLCV replicon producing the highest values. The BeYDV and BCTV systems reached their luminescence peak at 3 days postinfiltration, while TYLCV reached its maximum at 2 days postinfiltration. Notably, average basal luminescence in the absence of Rep was slightly higher for TYLCV and BCTV compared to BeYDV suggesting potential promoter activity conferred by regions within the IRs of TYLCV and BCTV (Figure 2B).

**Figure 2:**
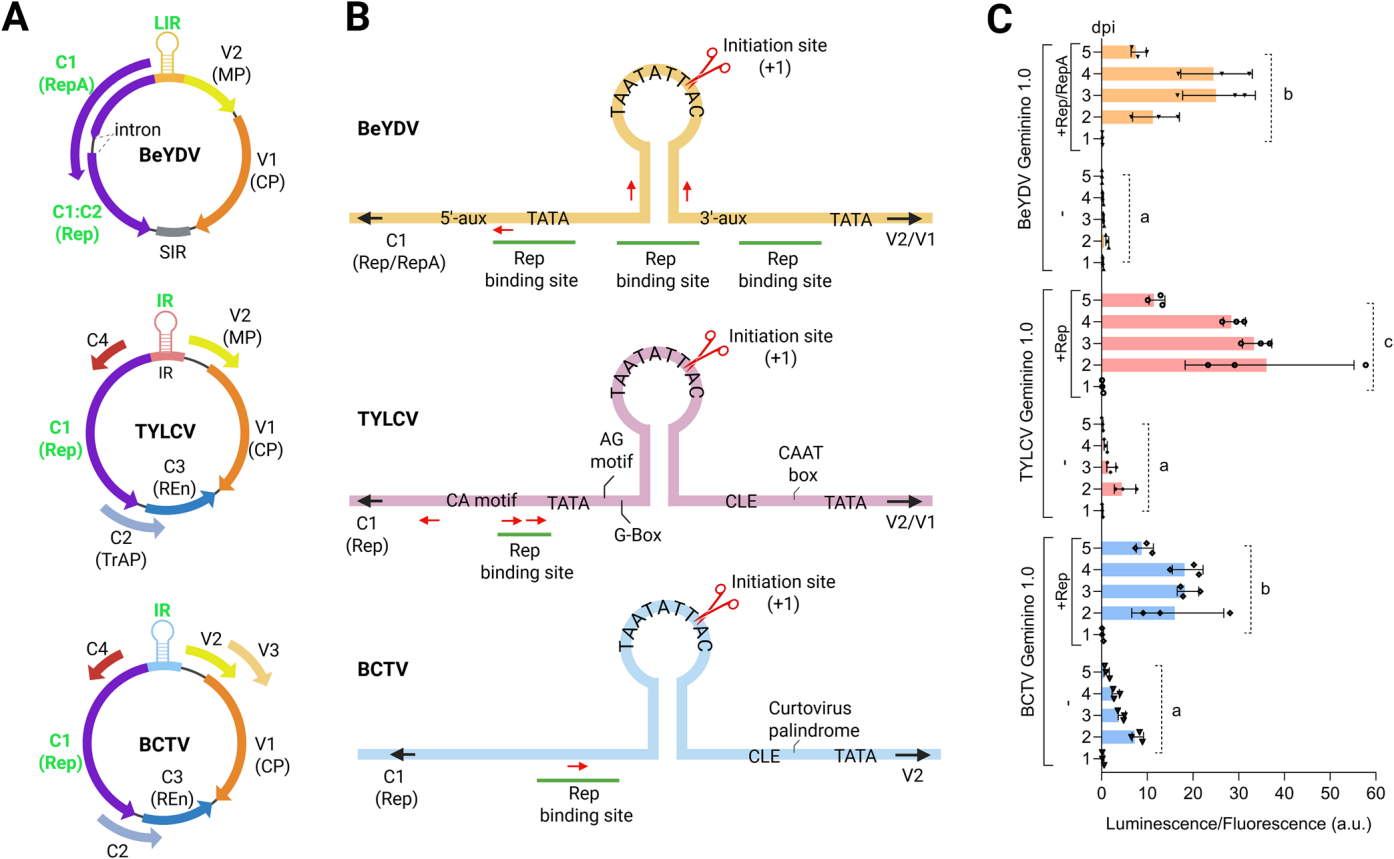
Geminiviral replication elements adapted from BeYDV, TYLCV, and BCTV genomes to create geminivirus-based systems for recombinant protein expression. (A) Schematic representation of BeYDV, TYLCV and BCTV genomes. Names of the elements involved in replication are displayed in green: Rep/RepA (C1/C2) for BeYDV, and Rep for TYLCV and BCTV (C1) and the (long) intergenic region (LIR/IR), which contains the origin of replication. (B) Detailed structure of the intergenic region for each virus. The replication origin (initiation site, + 1) where Rep nicks the DNA is marked by the red scissors. The Rep-binding sites are underlined, and iteron sequences are marked with red arrows. TATA box is required for transcription initiation, Auxiliary sequences (aux) and CA motifs enhance DNA replication, G-Box and AG motifs play a role in origin recognition, and conserved late element (CLE) enhances transcription. (C) Luminescence normalized by fluorescence values (luminescence/fluorescence arbitrary units) measured over 5 days (1–5) postinfiltration (dpi) for the different geminiviral vectors carrying HmS coexpressed with or without Rep­(/RepA) alongside the remaining genes of the bioluminescence pathway (H3H, Luz, CPH), the normalizer eGFP, and the silencing suppressor p19 (as depicted in Figure 1B). Each bar represents the mean of three independent leaf discs, each collected from a different leaf of the same plant (*n* = 3). Error bars represent standard deviation. Statistical analysis was performed using one-way ANOVA with Tukey’s multiple comparisons test (P-value ≤ 0.05), comparing the area under the curve (AUC) of each time-course. Bars sharing the same letter are not significantly different. The figure includes images from Biorender (biorender.com).

### Adaptation of the New Geminiviral Replicative Systems for Rep-Regulated Multiprotein Expression

One of the unique advantages of geminivirus-based systems over other RNA viral vectors for recombinant protein production is their ability to bypass replicative exclusion. This feature allows different geminivirus replicons to coexist within the same cell, making the system compatible with producing complex protein assemblies, where different subunits can be expressed from separate replicons. Taking advantage of the modularity of the system, we designed the so-called ‘COMBO’ configuration (Figure 3A). This configuration allows for the concatenation of multiple Geminino 1.0 replicons within a single T-DNA construct, enabling coordinated coexpression of several proteins. A newly optimized assembly syntax compatible with the GoldenBraid modular cloning system was implemented to allow a flexible assembly for the concatenation of an unlimited number of replicons. We then generated new COMBO constructs for all three geminiviral systems under study (BeYDV, TYLCV and BCTV) and employed them to coexpress three fluorescent reporter proteins (namely GFP, BFP, and dsRed) in via agroinfiltration (Figure 3A). In this case, we opted for fluorescent proteins instead of a luminescence-based reporter to enable the simultaneous detection of three distinct signals. This approach allowed us to assess whether the expressed proteins colocalized within the same cell, an analysis that would not be feasible using luminescence alone.

**Figure 3:**
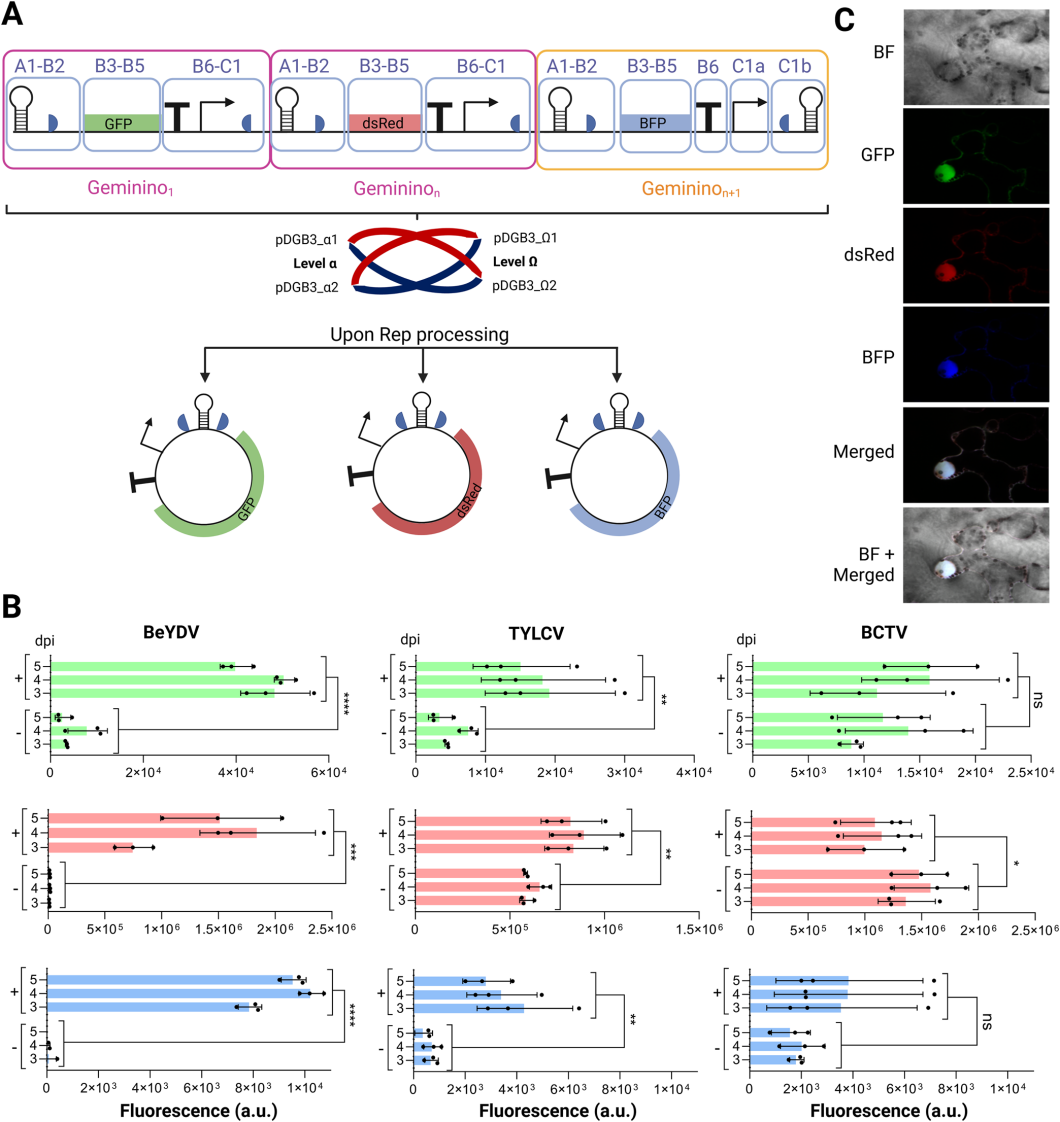
Design and performance of the COMBO configuration for multiprotein expression in . (A) Schematic representation of the COMBO configuration, enabling concatenation of multiple Gemininos 1.0 vectors within a single T-DNA construct. The assembly process utilizes an optimized cloning grammar compatible with the GoldenBraid system (represented by the GoldenBraid icon and its cloning vectors of level α and omega). Each DNA part has specific overhangs that give the identity to the part (A1-B2, B3–B5, etc.). *N* number of Gemininos can be combined in this configuration. LIRs are the long intergenic regions (LIRs) from BeYDV, SIR is the short intergenic region, half circles are the initial (1/2 intron) and the final (2/2 intron) segments of a unique intron, T is the transcriptional terminator, and the arrow indicates the transcriptional promoter. (B) Fluorescence expression levels of enhanced GFP (eGFP), BFP, and dsRed in leaves following agroinfiltration of COMBO configurations for BeYDV, TYLCV and BCTV systems and measured from 3 to 5 days postinfiltration (dpi). The ‘+’ symbol indicates coexpression with the Replication-associated proteins (Rep, or Rep/RepA in the case of BeYDV) of each system, while the ‘-‘ symbol indicates the absence of Rep­(/RepA). Each bar represents the mean of three independent leaf discs, each collected from a different leaf of the same plant (*n* = 3). Error bars represent standard deviation. Statistical analysis was performed using one-way ANOVA with Tukey’s multiple comparisons test (*P*-value ≤ 0.05), comparing the area under the curve (AUC) of each time-course. Asterisks indicate statistically significant differences (*P* ≤ 0.05); ‘ns’ denotes no significant difference. (C) Confocal microscopy images demonstrating colocalization of eGFP, BFP, and dsRed expressed via BeYDV COMBO system in the same leaf cell. BF stands for bright field. The excitation wavelengths for each fluorescence channel were: 488 for eGFP, 560 for dsRed, and 405 for BFP. Emission wavelength fluorescence range collected for eGFP, dsRed and BFP was 488–507, 558–583, 383–448, respectively. The figure includes images from Biorender (biorender.com).

Results showed tight regulation of expression by Rep/RepA in the case of the BeYDV system, showing negligible fluorescence values in the absence of Rep/RepA for each of the three reporters and strong fluorescence signal in the presence of Rep/RepA (Figure 3B). These results suggested a successful recircularization of the concatenated Gemininos 1.0 vectors and robust coexpression of the proteins. However, with the TYLCV and BCTV systems, basal fluorescence levels were notably higher compared to BeYDV, corroborating the previously observed basal activation of these new vectors. While the TYLCV COMBO still showed statistically significant differences with and without Rep, the BCTV COMBO results suggest that Rep might not effectively regulate recombinant protein expression. A combination of leaky expression and suboptimal BCTV Rep processing, consistent with previous luminescence-based observations, likely prevented the efficient recircularization of the Gemininos 1.0 vectors. For the BeYDV COMBO system, confocal microscopy analysis confirmed that eGFP, BFP, and dsRed proteins were coexpressed simultaneously in individual cells (Figure 3C). This highlights the capacity of geminivirus-based vectors to replicate and express multiple proteins in the same cell without interference.

### Exploration of Orthogonality between Geminiviral Replicative Systems

The development of new geminiviral replicons opens the interesting possibility of combining two or more systems in the same plant. The evaluation of the potential applications of such combinations requires previous analysis of their mutual interactions. To this end, we tested the expression levels conferred by all possible combinations of Rep proteins with the three Geminino 1.0 vectors (Figure 4A). Bioluminescence from the hybrid bioluminescence pathway was used as a reporter, with expression levels measured at 2–4 days postinfiltration. Results showed that the BeYDV system displayed strict specificity, with luminescence observed only when paired with its own Rep/RepA, and no cross-activation was observed when combined with the Reps from TYLCV or BCTV (Figure 4B). In contrast, the TYLCV and BCTV systems demonstrated some level of cross-reactivity. Although low, luminescence was detected when TYLCV and BCTV vectors were combined with Rep’s from other systems, particularly in the case of BCTV. However, the baseline luminescence observed in the absence of Rep, as noted previously, likely confounds the interpretation, as leakiness allegedly due to IR promoter activity could account for the apparent lack of specificity. To further explore this issue, luminescence images were taken from leaves infiltrated with combinations of each geminino vector and Rep, as well as controls without Rep (Figure 4C). These images confirmed the presence of significant leakiness in the TYLCV and BCTV gemininos, which appeared to be the primary cause of the observed nonspecific luminescence.

**Figure 4:**
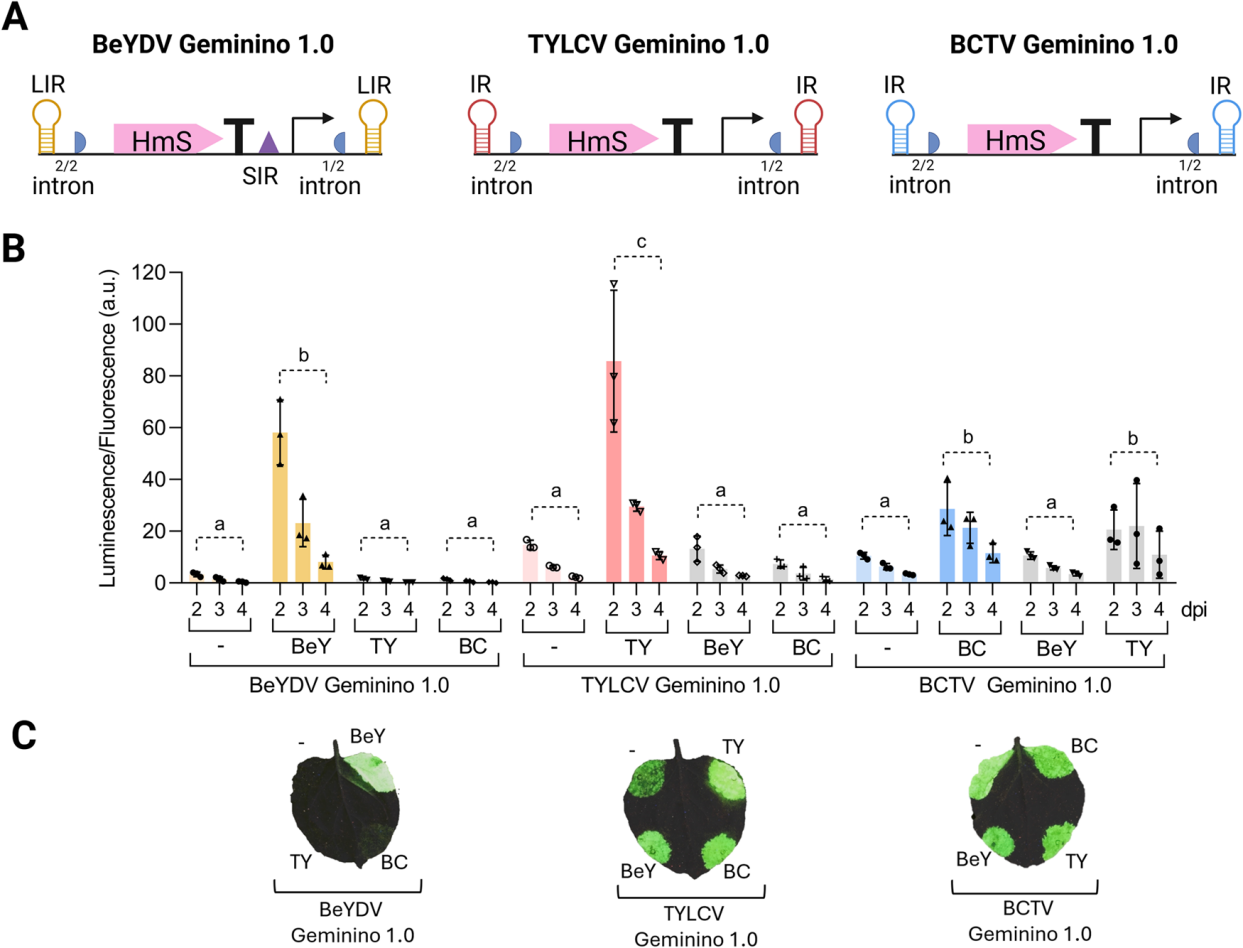
Cross-compatibility of replicative systems and hybrid Geminino 1.0 vectors. (A) Schematic representation of the Geminino 1.0 vectors carrying the hispidin synthase HmS for each geminiviral system (BeYDV, TYLCV, and BCTV). LIR/IR are the (long) intergenic regions (IR/LIRs), SIR is the short intergenic region, half circles are the initial (1/2 intron) and the final (2/2 intron) segments of a unique intron, T is the transcriptional terminator, and the arrow indicates the transcriptional promoter. (B) Luminescence normalized by fluorescence values (luminescence/fluorescence arbitrary units) resulting from the coexpression by agroinfiltration in of BeYDV, TYLCV, or BCTV Geminino 1.0 vectors carrying the hispidin synthase HmS with or without (−) the Replication-associated proteins (Rep, or Rep/RepA in the case of BeYDV) of BeYDV (BeY), TYLCV (TY), or BCTV (BC), the rest of the genes of the bioluminescence pathway (H3H, Luz, CPH), the normalizer eGFP, and the silencing suppressor p19 (as depicted in Figure 1B). Luminescence values were collected over 2–4 days postinfiltration (dpi). Each bar represents the mean of three independent leaf discs, each collected from a different leaf of the same plant (*n* = 3). Error bars represent standard deviation. Statistical analysis was performed using one-way ANOVA with Tukey’s multiple comparisons test (*P*-value ≤ 0.05), comparing the area under the curve (AUC) of each time-course. Bars sharing the same letter are not significantly different. (C) Luminescence imaging of leaves infiltrated with combinations of Geminino 1.0 vectors and the different Rep genes as described in (B). The figure includes images from Biorender (biorender.com).

### Optimization of TYLCV-Based Vectors

Owing to its higher activation and lower basal levels, we next decided to focus on the further optimization of the TYLCV vector, taking advantage of the bioluminescence reporter and modular cloning. To address the issue of unintended basal expression in the TYLCV geminino system, several alternative configurations were designed to reduce expression levels in the absence of Rep. These configurations focused on modifying or blocking any potential transcriptional regulation from the TYLCV IR sequence to minimize the undesirable expression of the gene of interest (Figure 5A). In the so-called Geminino 2.0, previously developed in our lab for BeYDV,^42^ the ATG start codon of the HmS gene was relocated downstream of the CDS to prevent early transcription from upstream promoter elements. We also created a Geminino 3.0, where a transcriptional terminator was inserted between the first IR and the HmS CDS to block any unintended transcriptional activity from the IR. Next, a Geminino 4.0 was developed, where a predicted TATA box within the IR, which was described to promote viral gene V2 expression, was mutated to prevent transcriptional leakage from these elements. Finally, in a Geminino 5.0, HmS was oriented in the antisense strand relative to the viral genome (complementary-sense strand), aimed at preventing any influence of the 3′ IR on HmS transcription. To preserve correct splicing in this orientation, the split intron comprising the first half intron downstream of the 5′ IR and the second half intron upstream of the 3′ LIR was also reversed, while the IR elements themselves remained in their native orientation. Each configuration was evaluated, with luminescence serving as a proxy for HmS expression. As can be observed in Figure 5B,C, Geminino 2.0 produced negligible basal luminescence in the inactivated state but also generated low luminescence levels when Rep was coexpressed, suggesting that this modification overly restricted gene expression, potentially impairing overall system functionality. Geminino 3.0 and 4.0 did not result in significant reductions in leakiness in comparison to Geminino 1.0. Both configurations continued to produce similar levels of luminescence to the original versions, indicating that the transcriptional terminator in Geminino 3.0 and the TATA box mutations in Geminino 4.0 were insufficient to fully prevent unintended transcription. By contrast, Geminino 5.0 yielded a significant reduction in basal expression for the TYLCV system while keeping luminescence levels comparable to the ones obtained with Geminino 1.0 (Figure 5B,C). The reverse orientation of the transcriptional unit substantially decreased luminescence, indicating that this approach effectively insulated HmS from the transcriptional influence of the TYLCV 3′ IR. The marked reduction in leakiness in the TYLCV Geminino 5.0 system highlights the potential of this approach for improving the specificity and control of geminino-based expression systems.

**Figure 5:**
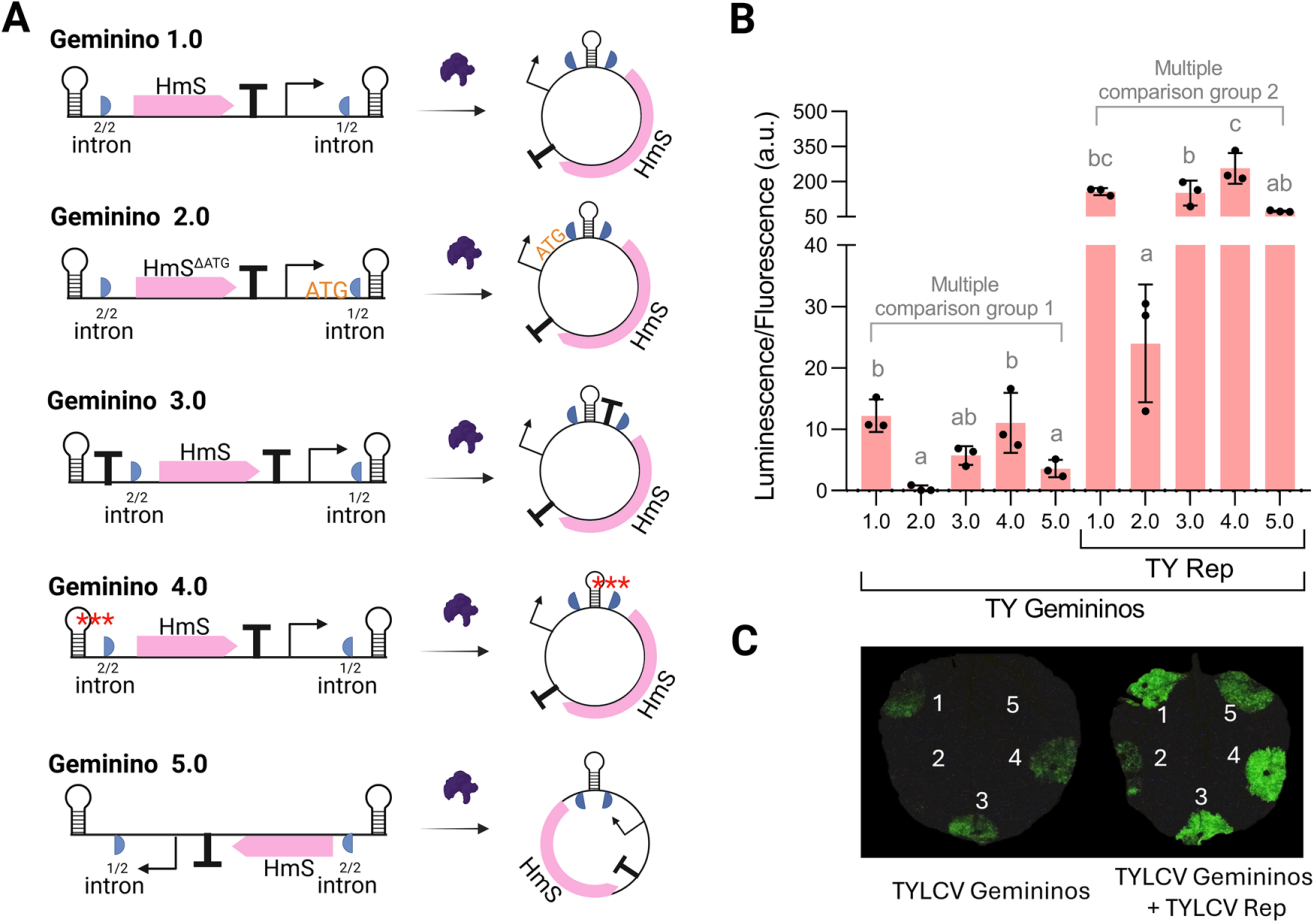
Optimization of TYLCV vector for low basal expression. (A) Schematic representation of Geminino vector configurations. The coding sequence of HmS is rearranged with the viral elements (intergenic regions, IR, represented by a steam-loop) to control precircularization expression levels. Red asterisks in Geminino 4.0 represent 3-nucleotides point mutation in a TATA box. (B) Luminescence normalized by fluorescence values (luminescence/fluorescence arbitrary units) resulting from the coexpression by agroinfiltration in of TYLCV Geminino vectors in (A) carrying the hispidin synthase HmS with the TYLCV Replication-associated protein (TY Rep), the rest of the genes of the bioluminescence pathway (H3H, Luz, CPH), the normalizer eGFP, and the silencing suppressor p19 (as depicted in Figure 1B). Luminescence values were collected at day 2 postinfiltration. Each bar represents the mean of three independent leaf discs, each collected from a different leaf of the same plant (*n* = 3). Error bars represent standard deviation. Statistical analysis was performed using one-way ANOVA with Tukey’s multiple comparisons test (*P*-value ≤ 0.05). Bars sharing the same letter are not significantly different. (C) Luminescence imaging of leaves infiltrated with combinations of Geminino vectors and TYLCV Rep as indicated in (B). The figure includes images from Biorender (biorender.com).

## Discussion

In this study, we expanded the palette of modular geminiviral tools incorporating new elements derived from BeYDV, TYLCV, and BCTV. Our aim was to open up the utility of geminiviral vectors beyond traditional roles in Molecular Farming, moving toward more sophisticated systems capable of regulating synthetic gene circuits and facilitating multiprotein expression. Modular tools enable the exchange and optimization of individual DNA parts, allowing for the fine-tuning of vector configurations. Earlier, Dusek et al.^35^ followed this modular approach to combine in a single hyperexpression system DNA elements coming from two previously described overexpression tools, namely the pEAQ vectors derived from Cowpea Mosaic Virus and the BeYDV replicon. Similarly, Neubauer et al.^36^ utilized the GoldenBraid modular cloning system to facilitate the optimization of a geminiviral vector. However, these studies have focused primarily on hyperexpression rather than regulation. In contrast, our study harnesses the modularity of GoldenBraid for regulatory purposes. By defining new standard overhangs for parts assembly, and by adapting DNA elements to these new overhangs, we have extended the standard “syntax” earlier proposed for modular cloning in plants^43^ to new uses in geminivirus biotechnology.

One of the key findings in this study is the suitability of using the fungal-derived bioluminescent pathway as a highly sensitive reporter system for tracking geminivirus-mediated protein expression. Previous studies have often relied on traditional reporters like GFP or GUS. However, these reporters are either destructive (GUS) or offer a limited dynamic range (fluorescence). While these limitations are less concerning when the goal is simply to maximize protein expression during transient expression, they become critical for more sophisticated goals such as genome integration and/or synthetic gene circuit engineering, where fine-tuned regulation and tight control of basal expression are required. Our results clearly demonstrate that HBP reporter is highly sensitive even when detected using consumer-grade cameras, enabling us to detect even minimal basal expression levels.

The HBP reporters allowed us to promptly assay several new geminiviral vector configurations. Specifically, in the case of TYLCV, new vector configurations involving alterations in the intergenic regions or reshuffling of transcriptional elements were immediately reflected in the luminescence intensity in both quantitative reads and imaging acquisitions. This responsiveness underscores the utility of the bioluminescence reporter for tracking even subtle adjustments in vector optimization. Additionally, we observed that the dynamics of luminescence correlated well with the typical timeline of geminiviral Rep-mediated expression, which usually peaks around day 2 or 3 postinfiltration, followed by a decline in expression thereafter. This is consistent with previously reported results in systems like CuBe,^31^ INPACT,^30^ and BeYDV replicon studies.^8^ What makes this particularly interesting is that our bioluminescence reporter measures the output of a metabolic pathway rather than direct protein expression, reinforcing the potential of geminiviruses as vectors for modulating entire metabolic pathways, as previously demonstrated by other studies, e.g., in the production of specific metabolites.^44^

Tight control over basal expression using BeYDV elements was already successfully demonstrated in CuBe^42^ and verified here using the HBP reporter. This could potentially be attributed to our use of a mild strain of the virus.^45^ By contrast, we encountered challenges in controlling basal expression when adapting TYLCV and BCTV vectors. The intergenic regions (IRs) of geminiviruses are known to contain promoter elements, including eukaryotic-like TATA boxes, which drive the expression of the viral genome in both virion-sense and antisense directions. As shown in Figure 2B, the IRs of TYLCV and BCTV contain several annotated promoter elements, including TATA and CAAT boxes.^5,46–50^ The presence of these elements could contribute to the leaky expression of the downstream HmS gene. To investigate this, we initially targeted what appeared to be the most critical element, the TATA box, for mutation. However, we observed no significant reduction in basal expression after its mutation. Further mutation of other elements may yield more promising results, as demonstrated by Fenoll et al.,^51^ who reported a 70% reduction in viral-sense promoter activity in MSV after mutating its GC-rich box. A high-throughput approach that introduces random mutations into the IR sequences may help identify the optimal combination for maintaining strong activation while minimizing basal expression. This strategy would be easy to test using the luminescence reporter system employed in our study.

In parallel, our results on designing TYLCV vectors highlight the trade-off in virus-derived expression systems between high expression output and tight regulatory control. This observation aligns with our previous findings using BeYDV vectors,^42^ where improved repression of basal activity often resulted in lower peak expression. Striking a balance between these two aspects is particularly critical in molecular farming, where achieving high recombinant protein yields must be weighed against minimizing unintended expression that may affect transgenic plant fitness. For TYLCV, we eventually arrived at a configuration, Geminino 5.0, that minimized basal expression while retaining high inducibility. In this design, the CDS is in reverse orientation, thus skipping any promoter activity from the IR located in the 5′end. Our results are in line with the transcriptomic analysis of TYLCV, showing higher transcriptional activity from the virion-sense strand.^52^ Similarly, in BCTV, the most highly expressed genes are V2 and V3 encoded in the virion-sense strand.^53^ However, despite the success of TYLCV Geminino 5.0, this configuration was unsuccessful for BCTV (data not shown). Neubauer et al.^36^ recently reported that the inclusion of a foreign promoter upstream of a gene of interest is unnecessary in BCTV-based vectors, as the IR itself acts as a stronger promoter than the 35S promoter. BCTV is suggested to have evolved from a recombination event between mastreviruses and begomoviruses, as it contains elements related to both viral groups.^53,54^ This recombinant origin suggests that BCTV is highly permissive and adaptable, which may make it difficult to domesticate for Synthetic Biology applications. The presence of elements from both mastreviruses (e.g., BeYDV) and begomoviruses (e.g., TYLCV) may explain why basal expression levels are similarly high when BCTV is combined with either the Rep protein from TYLCV or BCTV itself. This observation contributes valuable insights into the field of virology, particularly regarding the cross-replication capabilities of geminiviruses. It has already been documented that when two geminiviruses coinfect the same plant, recombination and mixing of viral elements can occur.^55^ The high infectivity and wide host range of BCTV may come with the trade-off of reduced replication efficiency, as suggested by our experiments comparing the performance of BeYDV, TYLCV, and BCTV. When it comes to achieving maximum expression levels, TYLCV appears to be the most promising geminivirus-based system for use in , which makes sense given its adaptation to solanaceous plants and match with the results from Kim et al.^26^

One of the most interesting and probably underutilized features of geminiviruses is their ability to support the replication of multiple viral vectors simultaneously within the same cell. We demonstrated how this capability can be harnessed for multiprotein expression by employing BeYDV-based triple replicons. Previously, Huang et al.^56^ and Dusek et al.^35^ also demonstrated the utility of geminiviruses for coexpressing up to two proteins. Huang et al.^56^ showed robust expression of the heavy and light chains of an antibody using a single vector with tandemly arranged pro-replicons, along with the accumulation of replicating forms of separate DNA episomes in agroinfiltrated *N. benthamiana* leaves. Similarly, Dusek et al.^35^ explored the coexpression of two proteins, GFP and dsRed, but in their case, they simply positioned two transcriptional units one after the other, flanked by the LIR and SIR-LIR sequences. In this work, we went a step further by successfully coexpressing three proteins in a Rep-regulated manner. Additionally, we investigated the influence of transcriptional unit positioning within the vector configuration to determine if it impacted expression or replication. Our results showed there is no clear order-dependent effect on expression, which contrasts with the findings from Dusek et al.,^35^ where the transcriptional unit in the first position was less expressed. However, their study did not clarify whether this difference was due to transcriptional interference or replication issues. It is possible that in their system, where two standard transcriptional units were concatenated, the constitutive promoter of the first construct might have influenced the expression of the second gene, explaining the higher expression levels observed when the same protein was placed in the second position. While our COMBO triple-replicon system enables the coexpression of multiple proteins, challenges remain in developing configurations that prevent constitutive expression in the absence of the Rep protein, particularly for TYLCV and BCTV. In the case of TYLCV, it would be worth exploring the use of the COMBO configuration by concatenating Geminino 5.0 units rather than Geminino 1.0 to improve regulation.

In conclusion, this study expands the geminivirus toolbox for Plant Synthetic Biology, providing new insights into how geminiviral vectors can be optimized for controlled activation, multiprotein expression, and advanced reporter systems. A promising future direction would be the combination of BeYDV-based Geminino 1.0 and TYLCV-based Geminino 5.0, each controlled by distinct Rep expression inputs. This approach could pave the way for multi-input regulated circuits with replicative elements in plants, enabling more sophisticated and dynamic control of gene expression in plant biotechnology.

## Methods

### Cloning and Assembly of GoldenBraid Constructs

The DNA constructs utilized in this study were created using the GoldenBraid (GB) system, as described by Sarrion-Perdigones et al.^43^. The sequences for the constructs developed can be accessed at https://goldenbraidpro.com/ using the IDs listed in Tables S1 and S2 and also accessed at and 10.5281/zenodo.15855892. Table S1 contains GB level 1 and higher-level transcriptional units and modules used in this study. A separate list of level 0 parts used specifically for Geminino assemblies is provided in Table S2. Briefly, the DNA parts were cloned into the pUPD2 vector, validated by restriction analysis and sequencing, and then integrated into transcriptional units using cyclic restriction-ligation reactions. These units were further combined into multigenic constructs using BsaI (for α vectors) or BsmBI (for omega vectors) enzymes and T4 ligase. Constructs were transformed into TOP 10 cells using the Zymo Research Mix&Go kit, and all assemblies were confirmed via restriction analysis.

### Agroinfiltration Experiments

For transient expression, the final plasmids were transformed into strain EHA105. Bacterial cultures were grown for 16 h at 28° and then resuspended in infiltration buffer (10 mM MES, 10 mM MgCl_2_, 200 μM acetosyringone, pH 5.6) to a final OD_600_ 0.1. After a 2-h incubation, equal volumes of cultures were mixed when required for coexpression, and they were coinfiltrated with a syringe into the abaxial side of three fully expanded leaves of five-week-old plants. Plants were maintained in a controlled environment with 16 h light/8h dark at 24/20 °C.

### Luminescence and Fluorescence Measurements

For luminescence/fluorescence ratios measurements in luminescence assays, leaf discs (5 mm diameter) were collected 24 h after infiltration and placed upside-down in a white 96-well plate (greiner bio-one, #655074) with 300 μL distilled water per well. Luminescence and fluorescence were measured in a GloMax Multi-Detection System (Promega). For time courses, plates were incubated at 25 °C, under 16h light/8h dark conditions, and 60–70% humidity. Luminescence was measured with a 10 s integration time, and eGFP fluorescence using excitation 490 nm and emission 510–570 nm. Ratios of luminescence to fluorescence were calculated for each sample and time point.

For fluorescence measurement in the coexpression of eGFP, dsRed, and eBFP in the COMBO configuration, leaf discs (5 mm diameter) were collected on day 3 postinfiltration and placed upside-down in a black 96-well plate (Corning, #3792). Fluorescence was measured using a PerkinElmer Victor X2Microplate Reader. Excitation wavelengths were set at 485 nm for eGFP, 530 nm for dsRed, and 405 nm for eBFP. The emission detection for fluorescence was 510 nm for eGFP, 595 nm for dsRed, and 510 nm for eBFP.

### Luminescence Imaging of Leaves

Leaves were harvested 24 h postinfiltration and placed in Petri dishes containing water to maintain hydration. Bioluminescence images were captured in a dark room using a SONY α7s camera set to an exposure time of 30 s, ISO 51200, and an aperture of f/2.8. The same ISO and aperture were used for bright-field images. To overlay dark-bioluminescence and bright-field images, Paint.net software was used, applying a transparency level of 200 to the bioluminescence layer. Leaves remained in the dark room (at room temperature) until images were captured on days 2 or 3 postinfiltration.

### Confocal Imaging to Colocalize eGFP, dsRed, and BFP Proteins

Leaf discs (5 mm diameter) were harvested for imaging at 3 days postinfiltration. These sections were examined using a Zeiss LSM 510-META confocal laser scanning microscope. For fluorescence detection, an argon-ion laser emitting at 488 nm or 405 was used to capture eGFP and BFP, respectively, while a helium–neon laser at 560 nm captured dsRed fluorescence. The emission ranges were 383–448 nm for eBFP, 488–507 nm for eGFP, and 558–583 nm for dsRed. Image acquisition was managed through Zen software, and postimaging analysis was conducted using Fiji (ImageJ) software.

## Supplementary Material



## Notes

GB level 1 and >1 transcriptional units and modules used in this study. Sequences can be found at https://goldenbraidpro.com/ using the GB number or GB name. The sequences of all the plasmids generated in this study are available at 10.5281/zenodo.15855892. All plasmids generated in this study are publicly available through Addgene. Plasmids are also available upon request from the authors (Table S1); GB level 0 DNA parts used to build the different Geminino constructs based on BeYDV, TYLCV, and BCTV. Sequences can be found at https://goldenbraidpro.com/ using the GB number. All plasmids are publicly available through Addgene and also upon request (Table S2) (PDF)

## Abbreviations

BeYDV: bean yellow dwarf virus
TYLCV: tomato yellow leaf curl virus
BCTV: beet curly top virus
dsDNA: double-stranded DNA
SIR: short intergenic region
IR: intergenic region
LIR: long intergenic region
RCR: rolling-circle replication
RDR: recombination-dependent replication
Rep: replication-associated protein
FBP: fungal bioluminescence pathway
HBP: hybrid bioluminescence pathway
GB: GoldenBraid
pNOS: nopaline synthase promoter
CaMV 35S: Cauliflower mosaic virus 35S
eGFP: enhanced GFP protein
BFP: blue fluorescent protein
