# Supplementary material for: Bioluminescence-Driven Optimization of Geminivirus-Based Vectors as Tools for Plant Biotechnology
Source: ACS Synth Biol. 2025 Aug 4;14(8):3078–90. doi: 10.1021/acssynbio.5c00164 (PMC12362603; doi:10.1021/acssynbio.5c00164)
Supplement: Supplementary file 1 [file sb5c00164_si_001.pdf]

Supporting information for

Bioluminescence-driven optimization of geminivirus-based vectors as tools for plant biotechnology

Elena Garcia-Perez[1], Victor Vazquez-Vilriales[1], Marta Vazquez-Vilar[1], Araceli G Castillo[2], Karen S Sarkisyan[3], Rosa Lozano-Duran[4], Eduardo R Bejarano[2], Diego Orzaez[1]

[1]Instituto de Biología Molecular y Celular de Plantas, CSIC-UPV, Valencia, Spain

[2]Instituto de Hortofruticultura Subtropical y Mediterranea 'La Mayora', Universidad de Málaga–Consejo Superior de Investigaciones Científicas (IHSM-UMA-CSIC), Universidad de Málaga, Málaga, Spain

[3]Institute of Clinical Sciences, Faculty of Medicine and Imperial College Centre for Synthetic Biology, Imperial College London, London, UK

[4]Centre for Plant Molecular Biology (ZMBP), Eberhard Karls University, Tübingen, Germany

Table S1. GB level 1 and >1 transcriptional units and modules used in this study. Sequences can be found at https://goldenbraidpro.com/ using the GB number or GB name. All plasmids are publicly available through Addgene and also upon request.

GB number	Construct	Description
GB0107	Empty vector	Used as a stuffer construct to get the same infiltration DO for every *Agrobacterium* mix.
GB5326	BeYDV Geminino 1.0-HispS	Transcriptional unit for the expression of Geminino under the 35S promoter for HispS expression for the bioluminescent pathway. BeYDV LIR1:2nd intron + HispS CDS to replace HispS in bioluminescence pathway + Ter35S:SIR + p35S + NEW 1st intron:LIR1. Deconstructed replicon that needs BeYDV Replicase for circularization and, thus, replication. The intronic parts have AGGT for processing.
GB4929	BeYDV Geminino 1.0-PKS2	LIR1:2nd intron + CDS encoding PzPKS2 from Plumbago zeylanica to replace HispS in bioluminescence pathway + Ter35S:SIR + p35S + NEW 1st intron:LIR1.
GB4928	BeYDV Geminino 1.0-ASCL	BeYDV LIR1:2nd intron + CDS encoding PpASCL from Physcomitrella patens to replace HispS in bioluminescence pathway + Ter35S:SIR + p35S + NEW 1st intron:LIR1.
GB4927	BeYDV Geminino 1.0-HmS	BeYDV LIR1:2nd intron + CDS HmS from Hydrangea macrophylla (PKS3) to replace HispS in bioluminescence pathway + Ter35S:SIR + p35S + NEW 1st intron:LIR1.
GB5386	TYLCV Geminino 1.0-HmS	Geminino 1.0 TYLCV vector expressing HmS from Hydrangea macrophylla (PKS3)

GB5469	TYLCV Geminino 2.0-HmS	TYLCV Geminino 2.0-no ATG on CDS. TYLCV IR + 2nd half intron ICON MP + HmS w/o ATG + t35S + P35s + 1st half of intron from ICON MP + TYLCV IR.
GB5458	TYLCV Geminino 3.0-HmS	Geminino version where a T35S is placed between IR TYLCV and 2nd half intron to try to prevent leakiness.
GB5546	TYLCV Geminino 4.0-HmS	TYLCV Geminino holding an IR with 3 nucleotide mutations to omit TATA box (CaGTta) to create Geminino 4.0.
GB5538	TYLCV Geminino 5.0-HmS	TYLCV IR + (1st intron + p35S + t35S + HmS CDS + 2nd intron on the reverse complementary strand) + TYLCV IR. Geminino in c-sense, aka Geminino 5.0 or ninobravo.
GB5388	BCTV Geminino 1.0-HmS	BCTV IR:2nd intron + HmS CDS + Ter35S + p35S + 1st intron:IR BCTV in alpha2. Deconstructed replicon that needs BCTV Rep for circularization and thus replication.
GB3598	pNOS:BeYDV Rep/RepA	Transcriptional unit for constitutive expression of BeYDV Rep/RepA (C1:C2).
GB4527	pNOS:TYLCV Rep	Transcriptional unit for constitutive expression of TYLCV Rep.
GB4552	pNOS:BCTV Rep	Transcriptional unit for constitutive expression of BCTV Rep.
GB5422	BeYDV combo_eGFP-dsRed-eBFP	BeYDV Geminino_eGFP + Geminino_dsRed + Geminino_eBFP in combo configuration. BeYDV Rep releases each Geminino independently for replication.
GB5419	TYLCV combo_eGFP-dsRed-eBFP	TYLCV Geminino_eGFP + Geminino_dsRed + Geminino_eBFP in combo configuration. BeYDV Rep releases each Geminino independently for replication.
GB5420	BCTV combo_eGFP-dsRed-eBFP	BCTV Geminino_eGFP + Geminino_dsRed + Geminino_eBFP in combo configuration. BeYDV Rep releases each Geminino independently for replication.
GB4485	CPH-LUZ-H3H-EGFP-p19	Module for the constitutive expression of the N. nambi CPH, LUZ, H3H genes, EGFP and p19 fused to a SF in alpha2.

Table S2. GB level 0 DNA parts used to build the different Geminino constructs based on BeYDV, TYLCV, and BCTV. Sequences can be found at https://goldenbraidpro.com/ using the GB number. All plasmids are publicly available through Addgene and also upon request.

GB number	Construct	Description
GB4135	pUPD2_Ter35S:SIR	Terminator 35S + BeYDV SIR to create BeYDV Geminino. It has the grammar of a 3' UTR.

GB4136	pUPD2_p35S Geminino 1.0/3.0/4.0	Promoter 35S domesticated for Geminino 1.0/3.0/4.0, so for being half of a terminator in GB grammar.
GB4469	pUPD2_1st half intron:LIR1 for Geminino 1.0 BeYDV	First half of intron from ICON MP + BeYDV LIR1 for creation of Geminino 1.0. Domesticated for being the second part of a terminator in GB grammar.
GB4470	pUPD2_LIR1 BeYDV:2nd half intron for Geminino 1.0 BeYDV	BeYDV LIR1 + 2nd half intron ICON MP to create Geminino 1.0.
GB4474	pUPD2_t35S for Geminino TYLCV & BCTV	T35S for building geminino TYLCV & BCTV. GB grammar 3'UTR.
GB4475	pUPD2_IR TYLCV:2nd half intron	IR TLCV + 2nd half intron ICON MP to create Geminino 1.0 TYLCV.
GB4476	pUPD2_1st half intron:LIR TYLCV	1st half intron ICON MP + IR TYLCV to create Geminino 1.0 TYLCV.
GB4535	pUPD2_IR BCTV:2nd half intron	Intergenic Region (IR) of beet curly top virus (BCTV) homologous to IR from TYLCV (637 bp) and amplified from BCTV-Stanley M24597_X04144 + 2nd half intron ICON MP to create Geminino BCTV.
GB4536	pUPD2_1st half intron:IR BCTV	1st half intron ICON MP + Intergenic Region (IR) of beet curly top virus (BCTV) homologous to IR from TYLCV (637 bp) and amplified from BCTV-Stanley M24597_X04144 to create Geminino 1.0 BCTV
GB5004	pUPD2_T35S:SIR-P35S-intron for COMBO	T35S + BeYDV SIR + P35S + 1st half intron from ICON MP for creation of new BeYDV Geminino 1.0 in COMBO. Domesticated for being 3'UTR + TERM.
GB5152	pUPD2_BCTV_5’LIR	BCTV 5' LIR for the assembly of BCTV replicon constructs in a single multipartite reaction.
GB5153	pUPD2_BCTV_3’LIR	BCTV 3' LIR for the assembly of BCTV replicon constructs in a single multipartite reaction.
GB5154	pUPD2_TYLCV_5’LIR	TYLCV 5' LIR for the assembly of TYLCV replicon constructs in a single multipartite reaction. Used to build Geminino 5.0 next to GB5472.
GB5155	pUPD2_TYLCV_3'LIR	TYLCV 3' LIR for the assembly of TYLCV replicon constructs in a single multipartite reaction. Used to build Geminino 5.0 next to GB5472.

GB5377	pUPD2_T35S-p35S-intron COMBO	T35S + P35S + 1st half intron from ICON MP for creation of TYLCV/BCTV Gemininos COMBO.
GB5466	pUPD2_IR TYLCV:2nd intron for Geminino 2.0	GB5466. TYLCV IR + 2nd half intron ICON MP to create Geminino 4.0-no ATG on CDS. Domesticated for being a promoter without AATG OH, but AGGT for intron processing.
GB5467	pUPD2_1st intron:IR TYLCV for Geminino 2.0	GB5467. First half of intron from ICON MP + TYLCV IR to create geminino 4.0-no ATG on CDS. Domesticated for being the second part of a terminator in GB grammar. It includes TG for having ATG and TC for Ser.
GB5472	pUPD2_TU for Geminino 5.0	1st intron + p35S + t35S + HmS CDS + 2nd intron on the reverse complementary strand. When assembling this unit to geminiviral IRs, geminino in c-sense is formed (Geminino 5.0 or ninobravo).
GB5540	pUPD2_IR TY mut-2nd intron	TYLCV IR with 3 nucleotide mutations to omit TATA box (CaGTta)+ 2nd half intron ICON MP to create geminino 4.0.
GB5541	pUPD2_IR BC mut-2nd intron	BCTV IR with 3 nucleotide mutations to omit TATA box (TTcgTgTA)+ 2nd half intron ICON MP to create geminino 4.0.
GB5542	pUPD2_IR TYLCV:t35S:2ndIntron	IR TYLCV + T35S + 2nd half intron for TYLCV Geminino 3.0 or Geminivictor.
GB5543	pUPD2_IR BeYDV:t35S:2ndIntron	LIR BeYDV + T35S + 2nd half intron for BeYDV Geminino 3.0 or Geminivictor.
